# An Enhanced Detector for Vulnerable Road Users Using Infrastructure-Sensors-Enabled Device

**DOI:** 10.3390/s24010059

**Published:** 2023-12-21

**Authors:** Jian Shi, Dongxian Sun, Minh Kieu, Baicang Guo, Ming Gao

**Affiliations:** 1 School of Mechanical Engineering, Beijing Institute of Technology, Beijing 100081, China; jianshijim@bit.edu.cn (J.S.); 3120215243@bit.edu.cn (D.S.); 2 Department of Civil and Environmental Engineering, University of Auckland, Auckland 1010, New Zealand; minh.kieu@auckland.ac.nz; 3 School of Vehicle and Energy, Yanshan University, Qinhuangdao 066004, China; guobaicang@ysu.edu.cn; 4 College of Mechanical and Vehicle Engineering, Hunan University, Changsha 410082, China; 5 School of Vehicle and Mobility, Tsinghua University, Beijing 100084, China

**Keywords:** object detection, VRU detection, infrastructure-sensors-enabled engineering, model lightweight

## Abstract

The precise and real-time detection of vulnerable road users (VRUs) using infrastructure-sensors-enabled devices is crucial for the advancement of intelligent traffic monitoring systems. To overcome the prevalent inefficiencies in VRU detection, this paper introduces an enhanced detector that utilizes a lightweight backbone network integrated with a parameterless attention mechanism. This integration significantly enhances the feature extraction capability for small targets within high-resolution images. Additionally, the design features a streamlined ‘neck’ and a dynamic detection head, both augmented with a pruning algorithm to reduce the model’s parameter count and ensure a compact architecture. In collaboration with the specialized engineering dataset De_VRU, the model was deployed on the Hisilicon_Hi3516DV300 platform, specifically designed for infrastructure units. Rigorous ablation studies, employing YOLOv7-tiny as the baseline, confirm the detector’s efficacy on the BDD100K and LLVIP datasets. The model not only achieved an improvement of over 12% in the mAP@50 metric but also realized a reduction in parameter count by more than 40%, and a 50% decrease in inference time. Visualization outcomes and a case study illustrate the detector’s proficiency in conducting real-time detection with high-resolution imagery, underscoring its practical applicability.

## 1. Introduction

As reported by the World Health Organization’s Global Status Report on Road Safety [1], approximately 1.35 million individuals perish annually in road traffic accidents worldwide. Notably, over half of these fatalities involve Vulnerable Road Users (VRUs). In prior research, pedestrians and cyclists, particularly those on bicycles and motorcycles, have been categorized as VRUs. Hence, employing advanced sensors and algorithms for the perception of VRUs in intricate traffic scenarios is crucial for ensuring their protection. This endeavor not only contributes to VRU safety but also supports the development of intelligent traffic monitoring systems and enhances the environmental perception capabilities of connected vehicles.

The field of object detection, a crucial domain in computer vision, has experienced significant transformations with the advent and progression of deep convolutional neural networks (CNNs). Tracing its lineage from the seminal LeNet architecture [2] to the more advanced ResNet frameworks [3], the application of CNNs for feature extraction has emerged as the predominant paradigm in object detection methodologies. This evolution in object detection can be critically analyzed along two principal dimensions: (1) Algorithmic architecture: This dimension delineates the progression from the two-stage to the one-stage object detection algorithms. The two-stage algorithms, notably the R-CNN series [4,5,6], employ an initial phase of candidate region proposal followed by a refinement stage for object detection. In stark contrast, the one-stage algorithms, as epitomized by the YOLO series [7,8,9], integrate these steps into a singular, unified process, thereby optimizing for computational efficiency and speed. (2) Anchor utilization: The concept of anchors or predefined bounding boxes has been a cornerstone in object detection. The Fast R-CNN typifies the anchor-based approach, utilizing these anchors to hypothesize potential object locations. Conversely, anchor-free algorithms, such as CenterNet [10], represent a paradigm shift towards detecting objects via key points, thereby streamlining the detection process and potentially enhancing accuracy. Moreover, the advent of specialized method-ologies such as SSD [11] has addressed the challenges inherent in small object detection, a sub-domain marked by the paucity of spatial information. The recent introduction of the Visual Transformer [12] marks a departure from conventional CNN-based approaches. By applying self-attention mechanisms to treat images as sequential data, this method offers a novel approach to capturing global dependencies within images, thereby broadening the horizons of object detection capabilities.

**Figure 1:**
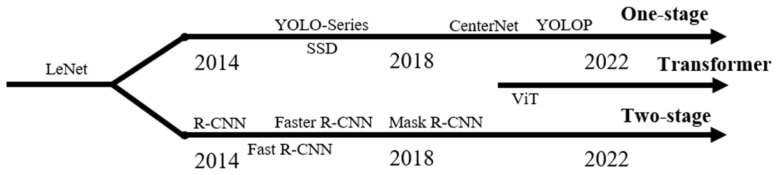
Development framework of classical object detection algorithms based on deep neural networks.

From the preceding summary of object detection (as shown in Figure 1), it is evident that object detection, rooted in computer vision and representing a form of advanced perception technology, is highly suitable for the perception of VRUs.

In the field of VRU perception, the research usually includes object detection, tracking and trajectory prediction of pedestrians and cyclists. In more detail, Nikhil et al. [13] used the YOLO algorithm to detect triple riding and speed violations on two-wheelers. Wang et al. [14] developed a cohesive framework for both detection and tracking, facilitating precise pedestrian detection. Chandan et al. [15] presented a fast and efficient approach to VRU detection and pose estimation for real-time AD applications. Kheireddine et al. [16] proposed a radar and camera fusion framework for reliable VRU detection, tracking and classification, the low detection rate of the camera in bad weather conditions is compensated by the radar detection. For a specific VRU category, like pedestrian, there are some studies for pedestrian attributes, characteristics, and low computational capability frameworks [17,18,19].

Infrastructure upgrades have facilitated the deployment of high-resolution visual sensors in intelligent monitoring [20,21,22], enabling advanced algorithms to process video and image data for more precise detection of VRUs. In particular, the link with the Internet of Things has made edge computing a mature tool [23,24,25]. However, this advancement also demands improved perceptual efficiency. Unlike traffic monitoring systems that operate offline, the accurate online identification of VRUs on edge infrastructure-sensors-enabled devices is imperative. Consequently, models designed for low computing power platforms must be sufficiently lightweight to guarantee real-time performance. Several studies have implemented methods to reduce model complexity in this domain, including pruning, distillation, and quantization, aiming to develop models that are more readily deployable on mobile devices. In more detail, Dai et al. [26] developed a YOLO-DS algorithm, leading to the proposal of an intelligent traffic object detection algorithm optimized for mobile platforms and this algorithm demonstrated notable performance on the Honor V20. Lan et al. [27] introduced a knowledge distillation framework that incorporates scale instances and teacher adaptation, specifically tailored for lightweight object detection. This approach yielded favorable outcomes on various public datasets. Song et al. [28] optimized the YOLOv5 algorithm using replacement backbone networks and reparameterization, achieving real-time pedestrian detection on multiple intranet devices in intelligent video surveillance.

With the ongoing emergence of new transportation modes, including powered two-wheelers and tricycles, the definition of VRUs necessitates expansion. Although these vehicles do not fit into the traditional VRU category, their vulnerable status in complex traffic scenarios relative to motor vehicles like cars justifies their inclusion. Nonetheless, research in this area remains notably limited.

As mentioned above, we have identified three primary challenges: (1) The majority of studies have concentrated on pedestrians and cyclists. However, with the rising prevalence of powered two-wheelers and tricycles in complex traffic scenarios, there is a growing need to redefine the traditional concept of VRUs. This redefinition is essential for developing fine-grained VRU detectors, a topic that remains underreported. (2) In object detection, the small-scale attributes of VRU groups, such as in images, are often insufficiently considered. This oversight is particularly critical in intelligent traffic monitoring systems, where the rates of false positives and missed detections for these groups are notably high. (3) Regarding real-time detection, the research focusing on the implementation of high-resolution image algorithms and their deployment on mobile platforms with constrained computing capabilities is still not sufficiently explored.

In this paper, we propose an enhanced VRU detector for small target perception with high-resolution imagery in the infrastructure unit. The main contributions of this paper are three-fold:

Initially, the YOLOv7-tiny algorithm is optimized through extensive modifications to the backbone network, neck, and head structures. This optimization yields a substantial enhancement in detection accuracy while maintaining nearly identical parameter count.

Subsequently, the implementation of pruning algorithm effectively halves the model’s computational load and inference time, thereby significantly boosting detection efficiency.

Ultimately, the refined model is deployed on the Hisense platform, where it demonstrates exceptional performance across both public datasets and specific case studies.

The overall structure of this article can be described as follows. In Section 2, we will discuss in detail the approach we proposed for VRU detection, including adjustments to the backbone network, detection head and neck, and the use of pruning algorithms to further lighten the model. In Section 3, we will use the public dataset and the engineering dataset together with a large number of ablation experiments to prove the progressiveness of the algorithm. In Section 4, we will discuss the rationality and improvement space of the algorithm, and explain some limitations.

## 2. Methods

In this section, we present an enhanced VRU detector tailored for infrastructure-sensors-enabled visual perception. Our primary objective is to attain real-time and precise VRU detection capabilities, specifically designed for deployment on resource-constrained infrastructure computing units. To enhance feature extraction performance for small targets, we introduce an attention mechanism without additional parameters into a lightweight backbone network. Our chosen pipeline utilizes YOLOv7-tiny as a foundation. Furthermore, we employ a slim network neck and a dynamic head for our detector, in conjunction with a pruning algorithm, to effectively curtail the network’s parameter count and realize the overarching goal of achieving model lightweightness.

### 2.1. An Enhanced VRU Detector Overall

#### 2.1.1. Original YOLOv7 Framework

**Figure 2:**
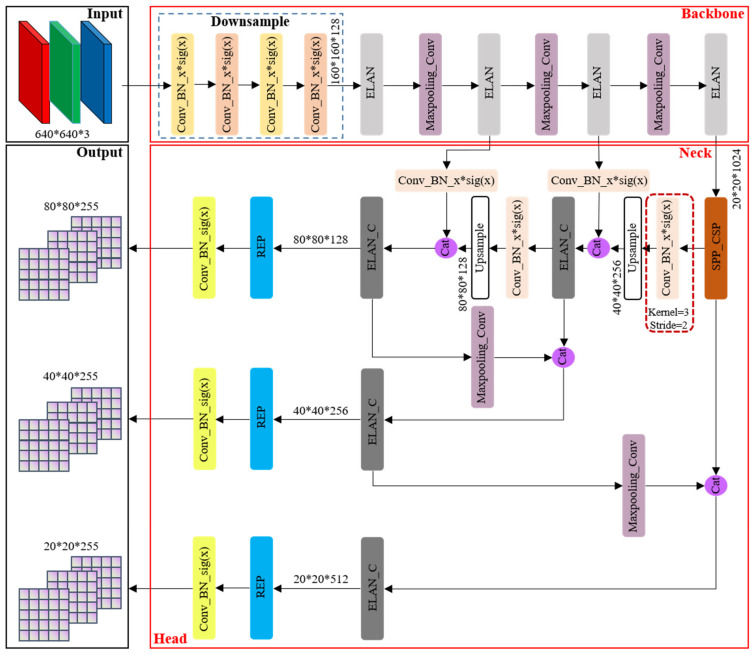
YOLOv7 original network structure framework.

As the flagship representative within the YOLO series of algorithms, YOLOv7 [29] amalgamates numerous advanced techniques and boasts several iterations, solidifying its position as a state-of-the-art (SOTA) algorithm in the realm of general object detection. However, it is noteworthy that the official documentation of YOLOv7 does not offer a direct schematic depiction of its network architecture. In light of this limitation, we have painstakingly constructed the original network structure framework based on the official open-source code, as illustrated in Figure 2.

**Figure 3:**
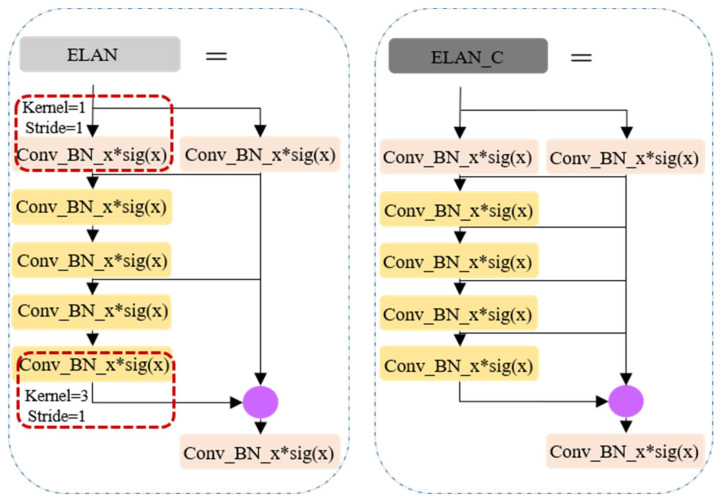
ELAN-like structure.

The principal advancements in YOLOv7, when juxtaposed with its predecessor YOLO versions, predominantly manifest in the following three key aspects: (1) Reparameterization integration: YOLOv7 introduces the incorporation of model reparameterization into its network architecture, signifying a fundamental shift in its design philosophy. (2) ELAN-like structure and model scaling strategy (as shown in Figure 3): This iteration presents an efficient layer aggregation network (ELAN) architecture, accompanied by a robust model scaling strategy. These innovations are pivotal in enhancing the overall efficiency of the network. (3) Training process enhancements: Throughout the training process, YOLOv7 incorporates a repertoire of training techniques aimed at refining accuracy, thus further solidifying its position as a cutting-edge solution.

In the original YOLOv7 algorithm series, YOLOv7-tiny was introduced specifically for edge devices, boasting a lighter and more deployable architecture. However, in our initial experiments aimed at the real-time detection of VRUs in infrastructure units, its performance fell short of expectations, particularly when tasked with conducting real-time model inference on high-resolution video streams and imagery. This shortfall was particularly evident due to the relatively smaller size of VRUs in comparison to other traffic entities, such as vehicles, resulting in a notably elevated rate of missed detections.

#### 2.1.2. Overall Architecture for the VRU Detector

**Figure 4:**
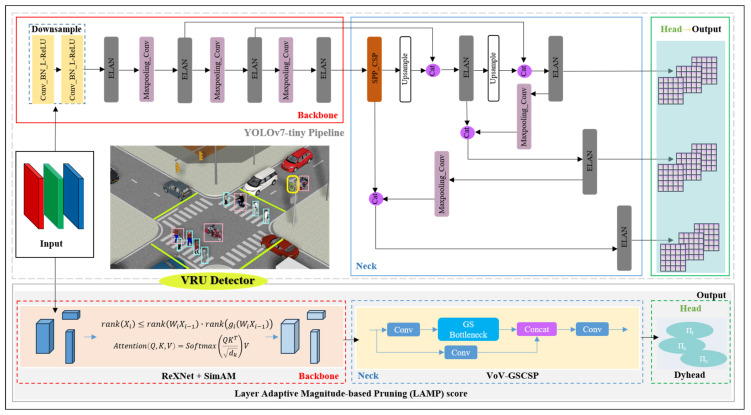
YOLOv7-tiny pipeline and overall architecture of our VRU detector.

For the analysis in Section 3.1.1, we established YOLOv7-tiny as the pipeline, as illustrated in Figure 4, with the goal of optimizing the backbone network, network neck, and head structure. To enhance its suitability for deployment on low-computing-power devices, we proceeded with pruning experiments on the refined network. This process culminated in the development of a real-time VRU detector specialized for perceiving small targets. The detailed architecture is depicted in Figure 4.

### 2.2. More Precise VRU Detection

#### 2.2.1. ReXNet-SimAM

(1) Rethinking the Backbone Network as ReXNet

The YOLOv7-tiny model maintains the established design ethos of the YOLOv7 series in its backbone network, utilizing an ELAN-like architecture to enhance the efficacy of feature extraction. Despite this adherence to a tried-and-tested framework, studies have suggested that such an architecture—beginning with three-channel inputs and methodically doubling the channel count at each stage up to the classification layer—may inadvertently create bottlenecks that constrain the flow of information within the network.

**Figure 5:**
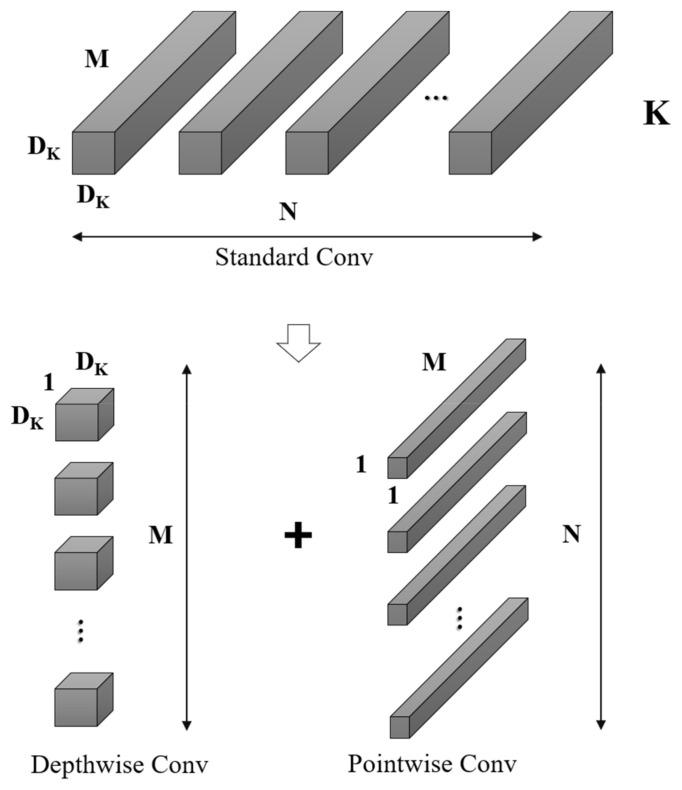
Standard convolutional decomposition in MobileNetV2.

In consideration of the necessity of implementing the VRU detection model on devices with limited computational capabilities, MobileNetV2 [30] emerges as a fitting choice. This model is commonly employed as a foundational network on mobile platforms for feature extraction, primarily due to its innovative utilization of depthwise separable convolutions to establish efficient neural networks. MobileNetV2 ingeniously splits the standard conventional convolution into a depthwise convolution and a pointwise convolution. This decomposition drastically curtails the number of parameters and the computational burden. Additionally, MobileNetV2 incorporates design principles from ResNet [3], employing a filter structure of *1 × 1* and *3 × 3* convolutions, and enhances feature propagation by applying shortcuts that facilitate the summation of outputs and inputs, as shown in Figure 5. Consider a given input feature map *F* with dimensions (*D_F_*, *D_F_*, *M*), a standard convolution kernel K of dimensions (*D_K_*, *D_K_*, *M*, *N*), and an output feature map *G* with dimensions (*D_G_*, *D_G_*, *N*). The computation of the standard convolution follows the formula presented in Equation (1).

(1) $$G_{k,l,n}=\underset{i,j,m}{\sum }K_{i,j,m,n}\cdot F_{k+i-1,l+j-1,m}$$

Assuming that the number of input channels is *M* and the number of output channels is *N*, the computational complexity can be expressed as follows

(2) $$\Omega =D_{K}\cdot D_{K}\cdot M\cdot N\cdot D_{F}\cdot D_{F}$$

The standard convolutional kernel, denoted as *K,* with dimensions (*D_K_*, *D_K_*, *M*, *N*), can be decomposed into depthwise convolution and pointwise convolution. In this decomposition, depthwise convolution is tasked with spatial filtering, characterized by dimensions (*D_K_*, *D_K_*, *1*, *M*), and yields output features of size (*D_G_*, *D_G_*, *M*). Subsequently, pointwise convolution, with dimensions (*1*, *1*, *M*, *N*), is utilized for channel transformation, culminating in the final output dimensions of (*D_G_*, *D_G_*, *N*). The computational process for depthwise convolution is detailed in Equation (3).

(3) $${\hat{G}}_{k,l,n}=\sum_{i,j,m}{\hat{K}}_{i,j,m,n}\cdot F_{k+i-1,l+j-1,m}$$

In Equation (3), $\hat{K}$ represents the depthwise convolution with a kernel size of (*D_K_*, *D_K_*, *1*, *M*). Here, each *m_th_* kernel uniquely corresponds to the *m_th_* channel of the input feature map *F*, generating the respective *m_th_* channel in the output feature map $\hat{G}$. The computational complexity for both depthwise and pointwise convolutions is computed as follows:

(4) $$\hat{\Omega }=D_{K}\cdot D_{K}\cdot M\cdot N\cdot D_{F}\cdot D_{F}+M\cdot N\cdot D_{F}\cdot D_{F}$$

Based on the above calculations, the total calculation amount has been reduced:

(5) $$\frac{D_{K}\cdot D_{K}\cdot M\cdot D_{F}\cdot D_{F}+M\cdot N\cdot D_{F}\cdot D_{F}}{D_{K}\cdot D_{K}\cdot M\cdot N\cdot D_{F}\cdot D_{F}}=\frac{1}{N}\cdot \;\frac{1}{D_{K}{}^{2}}$$

Depthwise separable convolutions are leveraged to significantly reduce parameter quantity. Addressing the network expression bottleneck identified previously, ReXNet [31]—a suite of network design strategies—has been introduced. To enhance MobileNetV2 using ReXNet principles, we focus on the following: (1) selecting optimal activation functions; (2) designing the network’s channels to increase progressively; and (3) incorporating additional expansion layers into the network. (For clarification, an ‘expansion layer’ is one where the number of output channels exceeds the number of input channels. Conversely, a ‘shrink layer’ is characterized by a lower number of output channels than input channels.) In adherence to the aforementioned principles, the enhanced structure of the MobileNetV2 network is delineated in Table 1 below, herein referred to as ReXNet-1.0x. It is noteworthy that the parameter quantity and FLOPs of the improved network remain broadly commensurate with those of its predecessor.

(2) An Attention Mechanism without Increasing Parameter Quantity

In Section 2.1.1, we identified that VRUs exhibit higher miss rates in high-resolution video streams during real-time model inference due to their relatively small scale compared to other traffic participants, like vehicles. To address this, we suggest integrating an attention mechanism within the feature-extracting backbone network to better detect small-sized targets, thus enhancing the precision of VRU detection. Traditional attention mechanisms often entail supplementary sub-networks such as SE (Squeeze-and-Excitation) [32] and CBAM (Convolutional Block Attention Module) [33], trading off parameter efficiency for improved accuracy. In contrast, our approach incorporates SimAM (Simple Attention Module), which augments the backbone network’s capability without escalating the parameter quantity.

**Figure 6:**
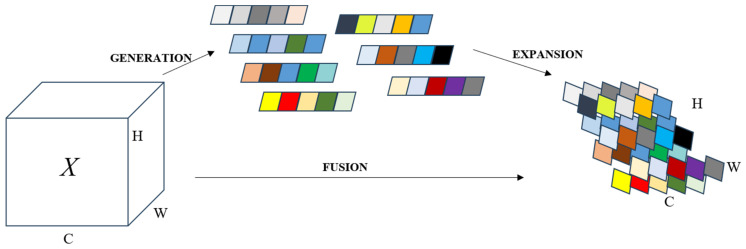
SimAM augments the backbone network’s capability without escalating the parameter quantity.

Contrasting with one-dimensional channel attention, two-dimensional channel attention, and spatiotemporal attention methods, SimAM [34] emerges as a unified three-dimensional weight attention module. Drawing insights from neuroscience research on neuronal behavior, SimAM introduces an energy function to identify salient neurons. It operates on the principle that lower energy corresponds to a higher disparity and significance of neuron t relative to its neighbors. By adhering to the tenets of attention mechanisms, this approach facilitates feature refinement without inflating the parameter count. Additionally, SimAM’s universal design allows for seamless integration with the backbone network, thereby bolstering feature extraction capabilities, as depicted in Figure 6.

#### 2.2.2. Dyhead for YOLOv7-Tiny

**Figure 7:**
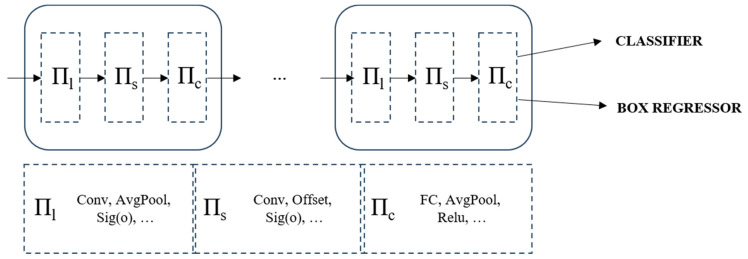
The principle and structure of Dyhead.

Typically, attention mechanisms are incorporated into the network’s neck block to augment its representational capacity. Given the considerable scale variations of VRUs in high-resolution imagery, we embed dynamic detection heads [35] within the YOLOv7-tiny architecture to enhance its performance. This improvement integrates multi-head self-attention mechanisms across the feature layers responsible for scale (*Π_s_*), spatial location (*Π_l_*), and task-specific channel (*Π_c_*) perception. The strategic amalgamation substantially elevates the representational prowess of the detection head. Crucially, it is achieved without incurring additional computational costs, aligning with our objective of developing a lightweight network for deployment, as shown in Figure 7. 

### 2.3. More Efficient VRU Detection

#### 2.3.1. VoV-GSCSP Block for Slim-Neck

**Figure 8:**
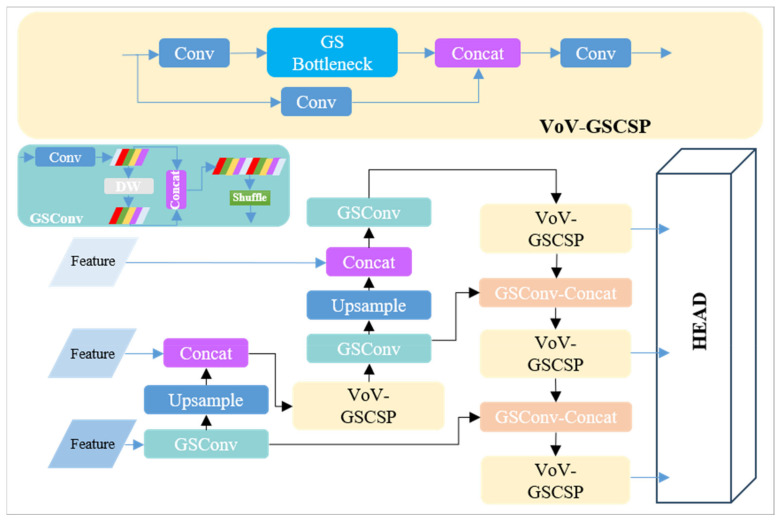
The structure of a slimmer ‘neck’, VoV-GSCSP. The DW module depicted in the figure refers to the depthwise convolution as described in Section 2.2.1.

To achieve a balance between network agility and the capability for real-time inference on resource-constrained roadside platforms, we refined the backbone and head parts as described in Section 2.2. Furthermore, we incorporated the VoV-GSCSP [36] Block into the YOLOv7-tiny pipeline, resulting in a slimmer ‘neck’. This configuration is represented in Figure 8.

#### 2.3.2. Lightweight Network Using LAMP

As a predominant technique for model compression [37,38,39], network pruning stands alongside the employment of efficient networks with minimal parameters. Pruning algorithms enable the removal of non-essential structures from the network, facilitating a reduction in size and capacity. These algorithms traditionally rely on manually-crafted heuristics or comprehensive hyperparameter optimization to establish levels of sparsity. However, the integration of dynamic heads, as detailed in Section 2.2.2, presents challenges for conventional pruning methods. To address this, we have adopted a novel global pruning importance metric based on the Layer Adaptive Magnitude-based Pruning (LAMP) score [40]. This metric, a normalized measure of weight magnitudes, accounts for the model-level l_2_ distortion induced by pruning, without necessitating hyperparameter tuning or intensive computations. Consequently, this approach streamlines training, enhancing simplicity and efficiency. The formula for the LAMP score is provided below:

(6) $$score\left(u;W\right)\;:\;\frac{{\left(W\left[u\right]\right)}^{2}}{\sum_{v\geq u}{\left(W\left[v\right]\right)}^{2}}$$

(7) $${\left(W\left[u\right]\right)}^{2}>{\left(W\left[v\right]\right)}^{2}\to score\left(u;W\right)>score\left(v;W\right)\;$$

We denote a depth-d feedforward neural network with weight tensors *W(1) W(d)* associated with each fully-connected or convolutional layer. It is assumed that each weight tensor is transformed into a one-dimensional vector. For each of these linearized vectors, the weights are sorted in ascending order based on a predefined index map. The LAMP score quantifies the relative significance of a given connection among all remaining connections within the same layer, particularly after connections of lesser weight magnitude in that layer have been pruned.

## 3. Experiments and Results

In this section, we present a series of ablation studies to verify the efficacy of our enhanced algorithm. Furthermore, we execute its engineering implementation on a low-computing-power platform, and through empirical cases, substantiate the considerable practical engineering value of our proposed VRU detector.

### 3.1. Setups and Datasets

#### 3.1.1. Experimental Environment

Our equipment and environment mainly included Intel(R) Xeon(R) Gold 6129 @ 2.30 GHz CPU, NVIDIA GeForce RTX 2080Ti*2 GPU (Santa Clara, CA, USA), and Ubuntu18.04.

**Figure 9:**
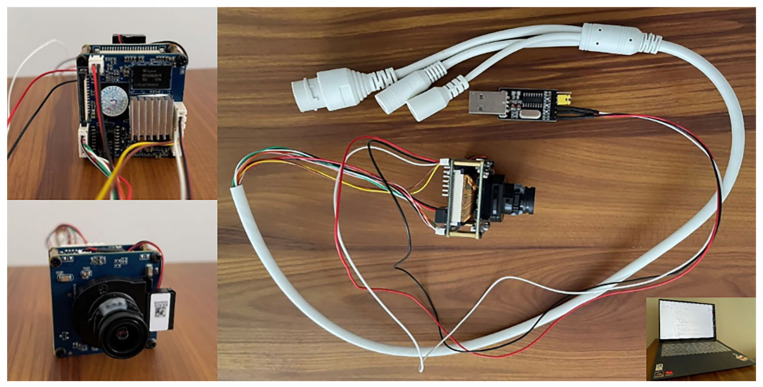
Hisilicon platform Hi3516DV300.

We transplanted the final models on a Hisilicon hardware platform, specifically, Hi3516DV300. The sensor model was a Sony IMX307 CMOS (Tokyo, Japan). The whole module computing capability was 1.0 Tops. Its cost was only one tenth of that of Nvidia Jetson NXⅡ. It is very suitable for simulating visual perception device in infrastructure units and is as shown in Figure 9.

#### 3.1.2. Training and Testing Datasets

**Figure 10:**
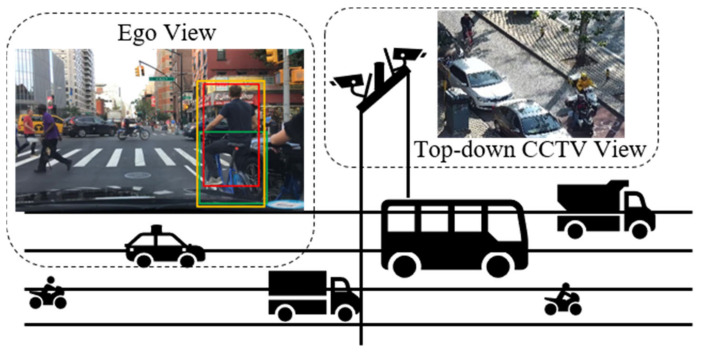
Image data is greatly affected by the acquisition angle. Different data sets do not label the same object. As shown in the Ego View block, Bdd100K labeled bicycles and cyclists separately, while KITTI labeled them as a whole.

In addition to datasets specifically dedicated to pedestrian detection and tracking, a number of public datasets for autonomous driving, such as KITTI [41] and BDD100K [42], also include annotations for VRUs. Analysis of these datasets’ annotations reveals varied interpretations of VRU classification, which are illustrated in Figure 10. 

**Figure 11:**
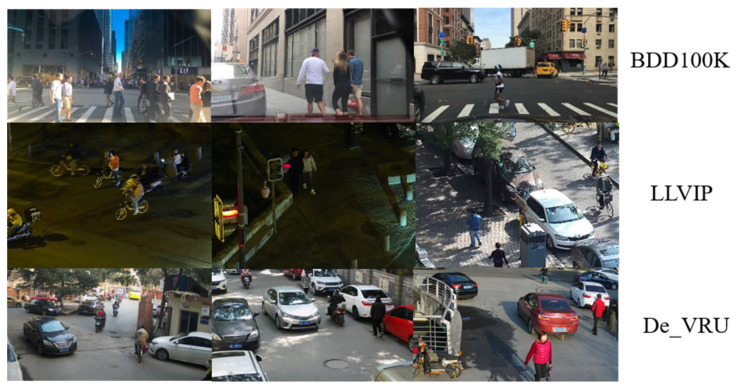
Sample imagery in BDD100K, LLVIP, and De_VRU.

Our objective was to detect a diverse array of VRU types with granular specificity. Consequently, we selected the BDD100K as the training set. Within BDD100K, VRU classification encompasses four categories: person, bike, motor, and rider. Given that the BDD100K dataset represents the ego-centric perspective of the primary vehicle, and our infrastructure unit necessitates a top-down bird’s-eye view akin to that of CCTV, we also selected the LLVIP public dataset as an additional dataset. However, the emergence of new transportation modes has expanded VRU categories in contemporary traffic settings to include tricycles and powered two-wheelers, whose rising involvement in traffic incidents warrants increased scrutiny. In response, we have collected and labeled imagery featuring additional types, resulting in the creation of an augmented dataset, designated as De_VRU. The DE_VRU dataset references annotations for the four VRU types included in BDD100K and further extends these by incorporating annotations for tricycles and powered two-wheelers (PTW), encompassing a total of six VRU categories. The dataset’s image resolution is set at 1920 × 1080. Details regarding these benchmarks are presented in Table 2, with sample imagery depicted in Figure 11.

### 3.2. Evaluation Metrics for Object Detection

We referred to recognized authoritative articles in the field [15,29] and determined evaluation metrics. The core evaluation metrics for object detection include accuracy, precision, recall, average precision (*AP*), and mean average precision (*mAP*), where *mAP* ranges from 0 to 1, with higher values indicating superior performance. This study presents *mAP* at a single intersection over union (*IoU)* threshold of 0.5 (*mAP@0.5*) and the mean value across a range of *IoU* thresholds from 0.5 to 0.95, in increments of 0.05 (*mAP@0.5:0.95*), providing a comprehensive assessment of detection precision. Additionally, model inference time on both GPU and CPU is utilized to gauge processing speed, with lower latencies denoting faster performance. We further incorporate commonly referenced indicators such as the number of parameters (*Params*), floating-point operations per second (*FLOPs*), frames per second (*FPS*), and the model’s storage footprint to provide a multifaceted evaluation of model efficiency. The formulas are as follows:

(8) $$mAP=\frac{\sum_{i=1}^{k}AP_{i}}{k}$$

(9) $$Params=n\times \left(h\times w\times c+1\right)$$

(10) $$FLOPs=H\times W\times n\times \left(h\times w\times c+1\right)$$

For the convolutional layer, we denote its dimensions as *h* × *w* × *c* × *n*, where *h* and *w* represent the height and width of the convolutional kernel, respectively, while *c* indicates the number of input channels, and *n* indicates the number of output channels. Here, *H* and *W* correspond to the height and width of the resulting output feature map, respectively.

### 3.3. Implementation Results

#### 3.3.1. Quantitative Analysis for Public Benchmarks

We employed the publicly available datasets BDD100K and LLVIP as benchmarks. The original YOLOv7-tiny was used as the baseline for conducting a series of ablation experiments on the enhanced backbone, neck, head components, and pruned network, as detailed in Section 3. These experiments were aimed at validating the efficacy of our proposed VRU detector.

The outcomes of the ablation study for network enhancement are presented in Table 3 and Table 4. Within these tables, ‘baseline’ refers to the model developed with the original YOLOv7-tiny. Other models denote variations where specific components of the baseline network are substituted, but all remaining elements are retained. For instance, ‘ReXNet1.0-SimAM’ signifies the substitution of the original YOLOv7-tiny backbone network with ReXNet1.0-SimAM, while preserving the original configurations of other network parts.

Ablation studies reveal that the combined use of ReXNet1.0-SimAM, VoVGSCSP, and Dyhead as optimized models substantially reduces *Model Size*, *FLOPs*, *Parameters*, and *Inference Time*. On the *mAP@0.5* metric for the testing set of the public dataset, these modifications result in an improvement of nearly 12%, an enhancement that represents a considerable increase in accuracy.

We used LAMP for pruning the enhanced networks of the backbone, neck, and head components, as detailed in Table 3 and Table 4, with YOLOv7-tiny serving as the baseline. The results post-pruning are presented in Table 5 and Table 6. Within these tables, ‘Baseline’ refers to the model developed with the original YOLOv7-tiny. ‘ReXNet1.0-SimAM+VoVGSCSP +Dyhead’ represents our improved network. ‘LAMP—1.5×’ and ‘LAMP-2.0×’ denote acceleration factors of 1.5 and 2, respectively. Upon reaching these predefined pruning thresholds, the network automatically transitions to the fine-tuning phase.

An analysis of Table 5 and Table 6 reveals that, relative to the enhanced networks presented in Table 3 and Table 4, networks pruned using LAMP exhibit notable improvements in model size, parameters, FLOPs, and inference time. While the network with 2× acceleration experienced a marked reduction in *mAP@50* and *mAP@0.5:0.95* metrics, the 1.5× accelerated network demonstrated robust performance on both the BDD100K and LLVIP public datasets. Compared to the baseline, this network achieved an improvement exceeding 12% in *mAP@50*, reduced the parameter count by over 40%, and halved the inference time. These results are particularly advantageous for deployment on low-computing-power platforms.

#### 3.3.2. Qualitative Analysis with Visualization and Case Study

**Figure 12:**
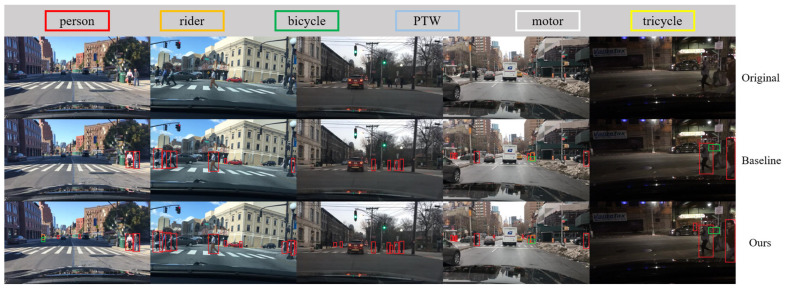
Visualization results of the models deployed on Hisilicon_Hi3516DV300 for BDD100K.

**Figure 13:**
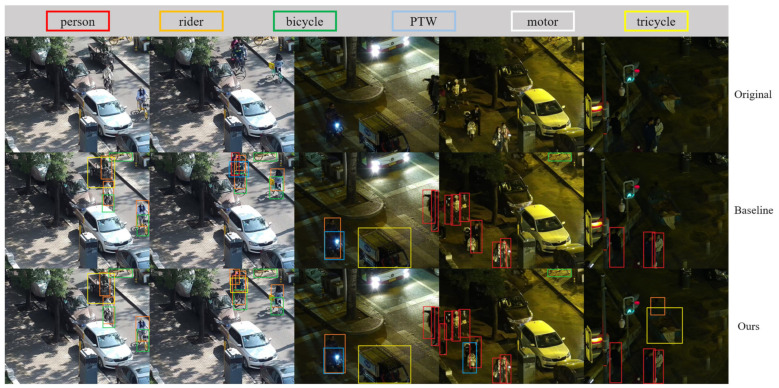
Visualization results of the models deployed on Hisilicon_Hi3516DV300 for LLVIP.

**Figure 14:**
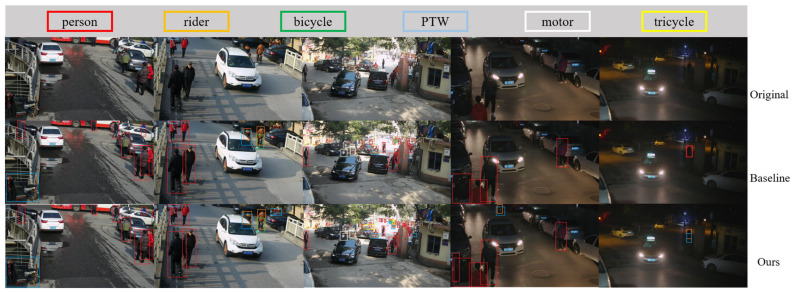
Visualization results of the models deployed on Hisilicon_Hi3516DV300 for De_VRU.

To evaluate the real-time performance and accuracy of our enhanced VRU detector, we utilized the baseline and LAMP-1.5x models, as referenced in Table 5 and Table 6, for pre-training. Subsequently, these models underwent further training and fine-tuning using the engineering dataset De_VRU. Additionally, we deployed the models independently on the Hisilicon_Hi3516DV300 platform. The visualization results on the publicly available datasets BDD100K and LLVIP, as well as the De_VRU dataset, are illustrated in Figure 12, Figure 13 and Figure 14 below.

The figure clearly illustrates that the baseline model of YOLOv7-tiny exhibits numerous missed and false detections in the test set. In contrast, our model consistently maintains robust detection performance in challenging conditions such as snowy and poor light environments. From the perspective of network structure, our network has strong feature extraction ability, especially for small targets, with good recognition performance. Additionally, when processing high-resolution images with dimensions of 1920 × 1080, our proposed VRU detector achieves a stable performance of 29FPS on the Hisilicon_Hi3516DV300 platform.

## 4. Conclusions and Discussion

To facilitate real-time detection from an infrastructure unit CCTV perspective, an enhanced VRU detector has been proposed. Initially, the YOLOv7-tiny model serves as the foundational pipeline, where the inclusion of a parameterless attention mechanism in the lightweight backbone network notably enhances the feature extraction efficiency for small VRU targets. The integration of dynamic detection heads further refines detection accuracy, while slimmer network necks reduce computational demands. This comprehensive enhancement on the optimized network, tested on the public datasets BDD100K and LLVIP, yielded a near 12% increase in *mAP@50* with minimal changes in model parameters and inference time. Subsequently, the network underwent further compression through the LAMP pruning algorithm, which, while preserving the nearly 12% increase in mAP@50, successfully halved both the parameter count and inference time. Finally, the pruned model was meticulously fine-tuned using an engineering dataset De_VRU and deployed on the Hisilicon_Hi3516DV300 platform. Operating on this platform, our enhanced VRU detector consistently achieved a detection speed of 29 FPS, with its effectiveness being empirically validated through visualization results and the case study.

Although our VRU detector has only been ported and tested on the Hisilicon platform, due to its simple network structure and easy deployment, it can also be applied to platforms such as Nvidia and Rockchips. Moreover, since we have fully considered the robustness of the model, it is applicable to both highways and complex urban traffic environments. In addition, as our work involves the issue of high-resolution images, it is essential to emphasize and explore the potential threats to privacy and the public. In our future research, we will use decentralized pretraining model weights and related techniques of federated learning to avoid the aforementioned issues as much as possible.

Despite the progress made, our study highlights two critical challenges warranting further investigation. First, the extensive range of VRU movement poses a significant challenge in ensuring safety through a singular camera setup. Future research should focus on enhancing cross-camera, real-time VRU recognition to address this issue. Second, while we have achieved a remarkably lightweight model through network optimization and pruning, facilitating real-time VRU detection on devices with limited computing power, the potential application of model quantization in practical engineering scenarios remains unexplored. This aspect presents an essential avenue for future research to further optimize model performance and efficiency.

## Figures and Tables

**Table 1:** Network structure of ReXNet-1.0x.

Input	Operator	Channels	Stride
224^2^ × 3	Conv3 × 3	32	2
112^2^ × 32	Bottleneck1	16	1
112^2^ × 16	Bottleneck6	27	2
56^2^ × 27	Bottleneck6	38	1
56^2^ × 38	Bottleneck6	50	2
28^2^ × 50	Bottleneck6	61	1
28^2^ × 61	Bottleneck6	72	2
14^2^ × 72	Bottleneck6	84	1
14^2^ × 84	Bottleneck6	95	1
14^2^ × 95	Bottleneck6	106	1
14^2^ × 106	Bottleneck6	118	1
14^2^ × 117	Bottleneck6	128	1
14^2^ × 128	Bottleneck6	140	2
7^2^ × 140	Bottleneck6	151	1
7^2^ × 151	Bottleneck6	162	1
7^2^ × 162	Bottleneck6	174	1
7^2^ × 174	Bottleneck6	185	1
7^2^ × 185	Conv1 × 1 Pooling7 × 7	1280	1
1^2^ × 1280	FC	1000	1

**Table 2:** Information about the benchmarks in this paper.

Dataset	Image Size (Pixels)	Acquisition View	Training Set	Testing Set	Annotations of VRUs Contained
BDD100K	1280 × 720	Ego View	70,000	20,000	4 (Person, Bike, Motor, Rider)
LLVIP	1280 × 1024	CCTV View	12,025	3463	1 (Person)
De_VRU (ours)	1920 × 1080	CCTV View	5000	1500	6 (Person, Bike, Motor, Rider, PTW, Tricycle)

**Table 3:** Performance of ablation experiments on the BDD100K testing set.

Model	Model Size (M)	*Params* *(M)*	*FLOPs* *(G)*	*mAP@0.5*	*mAP@0.5:0.95*	Inference Time (s)
Baseline	13.8	6.9	14.8	0.583	0.379	0.15
ReXNet1.0	13.1 ↑	5.6 ↑	14.6 ↑	0.531 ↓	0.342 ↓	0.13 ↑
ReXNet1.0-SimAM	13.2 ↑	5.6 ↑	14.6 ↑	0.569 ↓	0.361 ↓	0.13 ↑
VoVGSCSP	13.2 ↑	6.3 ↑	14.7 ↓	0.612 ↑	0.375 ↓	0.15 ↓
Dyhead	13.8 --	8.0 ↓	16.0 ↓	0.643 ↑	0.401 ↑	0.16 ↓
ReXNet1.0-SimAM+VoVGSCSP	**12.9** ↑	**5.1** ↑	**14.2** ↑	0.607 ↑	0.401 ↑	**0.12** ↑
ReXNet1.0-SimAM+ Dyhead	13.8 --	6.8 ↑	14.6 ↑	0.622 ↑	0.454 ↑	0.15 ↓
VoVGSCSP +Dyhead	13.7 ↑	7.4 ↓	15.4 ↓	**0.718** ↑	0.437 ↑	0.18 ↓
ReXNet1.0-SimAM+VoVGSCSP +Dyhead	14.1 ↓	7.1 ↓	15.2 ↓	0.709 ↑	**0.441** ↑	0.17 ↓

The data in the above table contain scaling time as a preprocessing time (resize input image to make its long side equal to 640) and inference time. The batch size is set at 32. The bold color represents the best results, ↑ and ↓ signify whether the corresponding metrics demonstrate an improvement or a decrease in comparison to the baseline, respectively.

**Table 4:** Performance of ablation experiments on the LLVIP testing set.

Model	Model Size (M)	*Params (M)*	*FLOPs* *(G)*	*mAP@0.5*	*mAP@0.5:0.95*	Inference Time (s)
Baseline	13.2	6.4	14.1	0.598	0.364	0.14
ReXNet1.0	**12.1** ↑	5.5 ↑	13.3 ↑	0.506 ↓	0.317 ↓	**0.12** ↑
ReXNet1.0-SimAM	12.2 ↑	5.5 ↑	13.3 ↑	0.610 ↑	0.357 ↓	0.13 ↑
VoVGSCSP	12.7 ↑	5.8 ↑	13.8↑	0.590 ↓	0.373 ↑	0.14 --
Dyhead	14.4 ↓	7.7 ↓	15.6 ↓	0.652 ↑	0.418 ↑	0.15 ↓
ReXNet1.0-SimAM+VoVGSCSP	12.5 ↑	**5.1** ↑	**12.9** ↑	0.589 ↓	0.404 ↑	0.13 ↑
ReXNet1.0-SimAM+ Dyhead	13.3 ↓	6.7 ↓	14.1 --	0.637 ↑	0.396 ↑	0.13 ↑
VoVGSCSP +Dyhead	13.9 ↓	7.1 ↓	14.3 ↓	0.719 ↑	**0.448** ↑	0.17 ↓
ReXNet1.0-SimAM+VoVGSCSP +Dyhead	13.7 ↓	6.9 ↓	14.7 ↓	**0.723** ↑	0.428 ↑	0.16 ↓

The data in the above table contain scaling time as a preprocessing time (resize input image to make its long side equal to 640) and inference time. The batch size is set at 32. The bold color represents the best results,↑ and ↓ signify whether the corresponding metrics demonstrate an improvement or a decrease in comparison to the baseline, respectively.

**Table 5:** Performance of pruning experiments on the BDD100K testing set.

Model	Model Size (M)	*Params (M)*	*FLOPs (G)*	*mAP@0.5*	*mAP@0.5:0.95*	Inference Time (s)
Baseline	13.8	6.9	14.8	0.583	0.379	0.15
ReXNet1.0-SimAM+VoVGSCSP +Dyhead	14.1 (100%)	7.1 (100%)	15.2 (100%)	**0.709** (−0.000)	**0.441** (−0.000)	0.17
LAMP—1.5×	7.7 (54.6%)	4.1 (57.0%)	10.2 (66.9%)	0.701 (−0.008)	0.420 (−0.023)	0.08
LAMP—2.0×	**5.9** (41.8%)	**3.0** (42.3%)	**7.6** (50.0%)	0.645 (−0.064)	0.405 (−0.036)	**0.07**

The data in the above table contain scaling time as a preprocessing time (resize input image to make its long side equal to 640) and inference time. The batch size is set at 32. The bold represents the best results.

**Table 6:** Performance of pruning experiments on the LLVIP testing set.

Model	Model Size (M)	*Params (M)*	*FLOPs (G)*	*mAP@0.5*	*mAP@0.5:0.95*	Inference Time (s)
Baseline	13.2	6.4	14.1	0.598	0.364	0.14
ReXNet1.0-SimAM+VoVGSCSP +Dyhead	13.7 (100%)	6.9 (100%)	14.6 (100%)	**0.723** (−0.000)	**0.428** (−0.000)	0.16
LAMP—1.5×	7.4 (54.0%)	3.9 (56.5%)	9.8 (66.9%)	0.721 (−0.002)	0.421 (−0.007)	0.08
LAMP—2.0×	**5.5** (40.1%)	**2.8** (40.6%)	**7.3** (50.0%)	0.695 (−0.028)	0.414 (−0.014)	**0.07**

The data in the above table contain scaling time as a preprocessing time (resize input image to make its long side equal to 640) and inference time. The batch size is set at 32. The bold represents the best results.

## Data Availability

The image sets used in the current study can be accessed from https://bupt-ai-cz.github.io/LLVIP (accessed on 5 October 2023) and https://bdd-data.berkeley.edu. (accessed on 5 October 2023).
